# Variation in the human soluble epoxide hydrolase gene and risk of restenosis after percutaneous coronary intervention

**DOI:** 10.1186/1471-2261-9-48

**Published:** 2009-10-08

**Authors:** Silke Kullmann, Priska Binner, Kirsten Rackebrandt, Andreas Huge, Georg Haltern, Mark Lankisch, Reiner Füth, Eberhard von Hodenberg, Hans-Peter Bestehorn, Thomas Scheffold

**Affiliations:** 1 Institute for Heart and Circulation Research, University of Witten/Herdecke, 44227 Dortmund, Germany; 2 Leibniz-Institute for Arteriosclerosis Research, University of Muenster, 48149 Muenster, Germany; 3 Heart Center Wuppertal Helios-Kliniken, 42117 Wuppertal, Germany; 4 Internal Medicine/Cardiology, Heart Center Lahr/Baden, 77933 Lahr, Germany; 5 Heart Center Bad Krozingen, 79189 Bad Krozingen, Germany

## Abstract

**Background:**

Restenosis represents the major limiting factor for the long-term efficacy of percutaneous coronary intervention (PCI). Several genetic factors involved in the regulation of the vascular system have been described to play a role in the pathogenesis of restenosis. We investigated whether the *EPHX2 K55R *polymorphism, previously linked to significantly higher risk for coronary heart disease (CHD), was associated with the occurrence of restenosis after PCI. The association with incident CHD should have been confirmed and a potential correlation of the *EPHX2 K55R *variant to an increased risk of hypertension was analysed.

**Methods:**

An overall cohort of 706 patients was studied: This cohort comprised of 435 CHD patients who had undergone successful PCI. Follow-up coronary angiography in all patients was performed 6 months after intervention. Another 271 patients in whom CHD had been excluded by coronary angiography served as controls. From each patient EDTA-blood was drawn at the baseline ward round. Genomic DNA was extracted from these samples and genotyping was performed by real-time PCR and subsequent melting curve analysis.

**Results:**

In CHD patients 6 month follow-up coronary angiography revealed a restenosis rate of 29.4%, classified as late lumen loss as well as lumen re-narrowing ≥ 50%.

Statistical analysis showed an equal genotype distribution in restenosis patients and non-restenosis patients (A/A 82.0% and A/G + G/G 18.0% versus A/A 82.1% and A/G + G/G 17.9%). Moreover, neither a significant difference in the genotype distribution of CHD patients and controls nor an association with increased risk of hypertension was found.

**Conclusion:**

The results of the present study indicate that the *EPHX2 K55R *polymorphism is not associated with restenosis after PCI, with incidence of CHD, or with an increased risk of hypertension and therefore, can not serve as a predictor for risk of CHD or restenosis after PCI.

## Background

Percutaneous coronary intervention (PCI) is a well established treatment strategy for patients suffering from symptomatic coronary heart disease (CHD). However, in numerous patients restenosis limits the clinical efficacy of this procedure. Restenosis represents a multifactorial process affected by a wide range of clinical, anatomic and procedural factors [1]. This includes, amongst others, the type of intervention such as PCI with or without intracoronary stent placement, type of stent, the nature of the affected vessels and the baseline characteristics of the patients [2-4]. Late lumen loss occurs due to a complex succession of processes, which have only been partially clarified so far. From this, vessel recoil, negative vascular remodelling and neointimal formation contributes to the development of restenosis [5,6].

Beside the classical risk factors and intervention procedures, several reports have demonstrated that genetic factors may be involved in the pathogenesis of restenosis after PCI. Here, gene products which engage in diverse physiological mechanisms, e.g. in the renin-angiotensin system, platelet aggregation, the inflammatory response, and smooth muscle cell proliferation, have been described to be associated with restenosis [7,8].

Several investigations have revealed regulatory functions of arachidonic acid metabolites within the vascular system [9,10]. Arachidonic acids are metabolized by cytochrome P450 epoxigenases to hydroxyeicosatetraenoic acids (HETEs) and epoxyeicosatrienoic acids (EETs), particularly in the kidney and vascular endothelium [11]. Among these eicosanoids, EETs represent potent vasodilators playing an important role in the regulation of vascular tone [11-13]. Additionally, EETs have anti-inflammatory properties [14,15], inhibit platelet aggregation [16] and promote fibrolysis [17,18].

The degradation of endogenous EETs to their corresponding diols, dihydroxyeicosatrienoic acids (DHETs), is catalyzed by soluble epoxide hydrolase (sEH) encoded by the *EPHX2 *gene located on chromosome 8p21-p12 [19,20]. Thus, the modulation of EET levels by sEH in the endothelium represents an important mechanism in the regulation of vascular functions. Several studies have validated an association between alterations in EET levels by the modulation of *EPHX2 *gene expression and cardiovascular risks in animal models and humans. For instance, sequence variation within the *EPHX2 *gene has been reported to reduce blood pressure in spontaneously hypertensive rats [21,22]. *EPHX2 *knockout mice exhibited significantly lower blood pressure than wild-type mice [23,24]. Furthermore, the effect of pharmacological inhibition of sEH in human blood vessels [25] as well as the association between *EPHX2 *sequence variants and risk of subclinical atherosclerosis has been published [26,27].

Previously, a study reported the correlation of the *K55R *single nucleotide polymorphism (rs41507953) within the *EPHX2 *gene with CHD in Caucasians [28]. The authors analysed a total of ten polymorphisms in coding and non-coding regions of *EPHX2*, which were selected based on their previously published functional relevance in vitro [29,30] and/or haplotype-tagging properties.

The *K55R *variant within the exon 2 of the *EPHX2 *gene was shown to be significantly more common among CHD cases than in the controls [28]. Additionally, carriers of the variant allele showed a higher epoxide hydrolase activity in vivo, indicated by lower plasma EET levels. No statistical differences were observed in the comparison of the genotype distribution in CHD cases and controls in the other analysed polymorphisms. Therefore, the findings of this study implicate the *EPHX2K55R *variant to be a genetic factor which increases the risk of CHD, even though this association has not been confirmed by further studies to date.

Due to the evident effects of *EPHX2 *in the regulation of vascular function and the association to CHD in the reported data, we assumed that the *K55R *variant may also be involved into the process of restenosis after percutaneous coronary intervention (PCI).

The main focus of the present project was to analyse whether the *EPHX2 K55R *variant allele is associated with the occurrence of restenosis within six months after in CHD patients. Moreover, we studied the distribution of genotypic and allelic frequencies of the *EPHX2 K55R *polymorphism in CHD patients and controls in order to confirm the association of this genetic variant with incident CHD and to analyse a potential correlation with increased risk of hypertension.

## Methods

### Study Population

Patients aged 35-80 years from Central Europe with symptomatic coronary heart disease who had undergone primary successful PCI of a native coronary artery were included into the study as described previously [31]. This multicenter, placebo-controlled study was designed to assess the effect of the calcium channel blocker Verapamil on restenosis after intervention. Successful intervention was defined by residual stenosis < 30% on visual estimation or desired position of stent. Follow-up coronary angiography was performed 6 months after PCI and the angiograms were quantitatively analysed.

Restenosis was determined through the measurement of the late lumen loss as well as the re-narrowing of the lumen ≥ 50%.

The study was approved by the local ethic committee and all patients gave written informed consent for further studies.

From this collective, a total of 435 patients, aged 35-79 years, were included into the present study to investigate the potential association between the *EPHX2 K55R *variant allele and rate of restenosis. Another 271 patients, in whom CHD had been excluded by angiography served as controls.

### Blood samples and DNA preparation

EDTA-blood samples were drawn from each patient at baseline ward round. Genomic DNA was extracted from 350 μl of these samples using the BioRobot EZ1 and the EZ1 blood extraction kit according to the manufacturer's instructions (QIAGEN; Hilden, Germany). DNA was quantified using the BioPhotometer (Eppendorf; Hamburg, Germany) and each sample was diluted to a final concentration of 25 ng/μl.

### PCR amplification and genotyping of *EPHX2 K55R*

PCR and detection of the genetic variants of *EPHX2 K55R *were performed on a LightCycler 480 instrument (Roche Applied Science; Mannheim, Germany) using primers and sequence specific hybridization probes designed and synthesized by TIB MOLBIOL (Berlin, Germany).

PCR was carried out in 96-well plates (Roche Applied Science; Mannheim, Germany) using 62.5 ng of genomic DNA as template in a final reaction volume of 10 μl.

The reaction mixture contained 0.5 μM of each primer (for: 5'-GTGTTTTCCAGAGGACTTCTGA, rev: 5'-GTGACTGCAATACTCCCATTAATA), 0.15 μM of SNP-specific hybridization probes (sensor: 5'-LC640-GCTTATGAAAGGAGAGATCACACT-PH; anchor 5'-GGGGACCAGAGGGTGCCACTACCC-FL), and 2 μl LightCycler 480 Genotyping Master Mixture (Taq DNA polymerase, reaction buffer, 15 mM MgCl_2_, and a dNTP mixture with UTP instead of dTTP) (Roche Applied Science; Mannheim, Germany).

The cycling program consisted of 10 minutes of initial denaturation at 95°C, followed by 40 cycles of denaturation at 95°C for 5 seconds (ramp rate 4.4°C/s), annealing at 55°C for 10 seconds (ramp rate 2.2°C/s), and extension at 72°C for 10 seconds (ramp rate 4.4°C/s). After PCR melting curves were generated by holding the reaction mixture at 95°C for 1 minute, lowering the temperature to 55°C for 30 seconds, holding it for 30 seconds at 45°C, followed by continuously heating to 80°C. Melting curve analyses were conducted by means of the LightCycler 480 software according to the manufacturer's instructions (Roche Diagnostics Inc.; Mannheim, Germany).

### Statistical analysis

Genotype distributions and allele frequencies of the *EPHX2 K55R *polymorphism in CHD patients and controls or the analysed subgroups were compared by chi-square test using a free program . For all data, the association was considered to be significant for p < 0.05.

Genotypes were tested for Hardy-Weinberg equilibrium among MI cases and controls using a chi-square test with one degree of freedom.

## Results

### Study population

A comparison of the baseline clinical characteristics and the procedural parameters of CHD cases and controls is presented in Table 1.

**Table 1:** Baseline characteristics of the study population

***Characteristic***	***CHD cases*** ***(n = 435)***	***Controls*** ***(n = 274)***
Age at recruitment	61 ± 8.9	59 ± 9.6
		
Men	365 (83.9)	129 (47.1)
		
BMI -- kg/m^2^	28 ± 3.5	27 ± 5.3
≥ 30 kg/m^2^	103 (23.7)	49 (17.9)
		
Medication		
Verapamil	220 (50.6)	--
Placebo	215 (49.4)	--
		
Event		
Intracoronary stent	362 (83.2)	--
Previous CABG	17 (3.9)	--
Previous PTCA	43 (9.9)	--
Previous MI	156 (35.9)	--
		
Cardiovascular risk factor		
Diabetes mellitus	54 (12.4)	22 (8.0)
Hypertension	279 (64.1)	139 (50.7)
Hyperlipidaemia	373 (85.7)	138 (50.4)
Current smoker	103 (23.7)	40 (14.6)
Former smoker	163 (37.5)	47 (17.2)
		
Family history of CHD	166 (38.2)	n/a
		
Restenosis	128 (29.4)	--

Data presented are number (%) of patients; plus-minus values are mean ± standard deviation. *BMI *body mass index, *CABG *coronary artery bypass grafting, *PTCA *percutaneous transluminal coronary angioplasty, *MI *myocardial infarction, *CHD *coronary heart disease, *n/a *not applicable.

The age of the CHD patients compared to the controls was approximately equal in both groups (61 ± 8.9 versus 59 ± 9.6). However, the proportion of male subjects was significantly higher in CHD patients than in the control group (83.9% versus 47.1%). As expected, patients suffering from coronary heart disease revealed a higher prevalence of hypertension, diabetes mellitus, hyperlipidaemia and smoking habit compared to the controls.

### *EPHX2 K55R *and restenosis

CHD cases were subdivided into two groups according to the occurrence or lack of restenosis at the 6 month follow up. For the further analysis, no differentiation was made between patients with late lumen loss and angiographic restenosis.

Over this period, 29.4% of the investigated 435 patients developed a restenosis.

The *EPHX2 K55R *genotype distribution was determined and compared between both groups, whereby A/A represented the wild-type, A/G the heterozygous and G/G the homozygous mutant genotype.

The distribution was 82.0% A/A and 18.0% A/G + G/G in patients with restenosis compared to 82.1% A/A and 17.9% A/G + G/G in patients without (p = 0.989) (Table 2). Hence, this analysis failed to show any significant influence of the *EPHX2 K55R *polymorphism on angiographic restenosis after PCI.

**Table 2:** *EPHX2 K55R *in restenosis and non-restenosis patients

	***CHD cases*** ***Restenosis*** ***(n = 128)***	***CHD cases*** ***No Restenosis*** ***(n = 307)***	***P value***
**Genotype**			
A/A	105 (82.0)	252 (82.1)	
A/G + G/G	23 (18.0)	55 (17.9)	0.989
			
**Allele frequencies**			
A	0.91	0.91	
G	0.09	0.09	

Data presented as absolute (%) genotype frequency and allele frequency.

Additionally, an alignment of the genotype distribution was conducted in subgroups of CHD patients who received verum (n = 220) and placebo (n = 215) to ensure an approximately equal distribution in these groups. With this analysis, any effects on the development of restenosis by random interactions between medication and genotype were excluded (Data not shown).

### *EPHX2 K55R *and CHD

In order to confirm the previously reported association of *EPHX2 K55R *with an increased risk of CHD the genotype and allele frequencies in 435 CHD patients and 271 controls with no pathological findings shown by coronary angiography, were analysed. The genotype distribution did not significantly (P > 0.1) deviate from the Hardy-Weinberg equilibrium (Table 3).

**Table 3:** *EPHX2 K55R *genotype distribution in incident CHD and early-onset CHD cases

	***CHD*** **(n = 435)**	***Controls*** **(n = 271)**	***HW***	***P value***	***CHD*** ***≤55 years*** **(n = 117)**	***Controls*** ***≤55 years*** **(n = 91)**	***P value***
**Genotype**							
A/A	357 (82.1)	219 (80.8)			92 (78.6)	75 (82.4)	
A/G + G/G	78 (17.9)	52 (19.2)	0.527	0.675	25 (21.4)	16 (17.6)	0.496
							
**Allele frequencies**							
A	0.91	0.90			0.89	0.91	
G	0.09	0.10			0.11	0.09	

Data presented as absolute (%) genotype frequency and allele frequency. HW: P-value for Hardy-Weinberg equilibrium test.

As demonstrated in table 3 the distribution of genotypes was 82.1% A/A and 17.9% A/G + G/G in CHD cases versus 80.8% A/A and 19.2% A/G + G/G in the control group (p = 0.675). Consequently, no statistically significant differences in the distribution of genotypes of CHD cases compared to controls could be observed. Thus, the association of the *K55R *allele variant with CHD could not be confirmed in the examined study population.

Additional analysis was performed to investigate the potential relevance of *EPHX2 K55R *to the early-onset of CHD. Therefore, male patients with early-onset CHD, defined as clinical CHD occurring at the age of ≤ 55 years and matched controls were divided into groups and statistical analysis was performed. This analysis also showed no significant difference in the genotype distribution and allele frequencies of CHD cases and controls according to the age of the subjects (Table 3).

### *EPHX2 K55R *and risk of hypertension

To investigate the correlation between increased risk of hypertension and the *K55R *variant allele subgroup analysis was conducted. The *EPHX2 K55R *genotype distribution in patients with raised blood pressure was 83.5% A/A and 16.5% A/G + G/G in CHD cases versus 83.2% A/A and 16.8% A/G + G/G in the control group (Table 4). Therefore, the *K55R *variant allele was not significantly associated with an increased risk of hypertension (p = 0.938).

**Table 4:** *EPHX2 K55R *genotype distribution in subgroups of cardiovascular risk factors

	***CHD cases***	***Controls***	***P value***
**Hypertension**	*n = 279*	*n = 137*	
A/A	233 (83.5)	114 (83.2)	
A/G + G/G	46 (16.5)	23 (16.8)	0.938
			
**Male**	*n = 365*	*n = 126*	
A/A	299 (81.9)	106 (84.1)	
A/G + G/G	66 (18.1)	20 (15.9)	0.574
			
**Diabetes mellitus**	*n = 54*	*n = 20*	
A/A	44 (81.5)	17 (85.0)	
A/G + G/G	10 (18.5)	3 (15.0)	0.724
			
**Hyperlipidaemia**	*n = 373*	*n = 133*	
A/A	304 (81.5)	106 (79.7)	
A/G + G/G	69 (18.5)	27 (20.3)	0.649
			
**Current Smoker**	*n = 103*	*n = 39*	
A/A	87 (84.5)	31 (79.5)	
A/G + G/G	16 (15.5)	8 (20.5)	0.480

Data presented as absolute (%) genotype frequency and allele frequency in subgroups for cardiovascular risk factor.

None of the other cardiovascular risk factors was significantly associated with a higher frequency of the risk variants A/G and G/G of the *EPHX2 *polymorphism in CHD patients examined by means of subgroup analysis (Table 4).

## Discussion

Several polymorphisms within the *EPHX2 *gene have been reported to influence the enzyme activity and stability of soluble epoxide hydrolase causing alterations in EET levels [29,30,32]. Therefore, genetic variations in *EPHX2 *may affect the beneficial properties of EETs on the vascular system and thus, could be linked to risks for cardiovascular events [26,27].

Based on the previously reported association of the *EPHX2 K55R *polymorphism with incident CHD in an American study population of Caucasian origin [28], we investigated whether the *K55R *variant allele was associated with restenosis in patients who had undergone primary successful PCI. Our results demonstrate no significant correlation between the *EPHX2 *variant and the occurrence of restenosis within 6 month after PCI investigated by means of subgroup analysis. None of the known risk factors for restenosis, such as diabetes mellitus or hypertension, could be identified as the cause for the occurrence of restenosis in 29.4% of our patient cohort. We assume that late lumen loss in the studied patient cohort is mainly caused by hyperplasia of the vascular endothelium [33,34]. The findings in the present study indicate that an involvement of the EPHX2 K55R variant on this mechanism can largely been excluded.

As no association between *K55R *and restenosis could be observed, we assumed that the previously reported association of the *EPHX2 K55R *polymorphism with incident CHD could be validated in the present study. However, we could not observe this association with the incidence of CHD in the present study.

It was shown that the association of polymorphisms within the *EPHX2 *gene with cardiovascular risks strongly depends on the ethnical background of the analysed populations [26,35]. Indeed, the reported association could only be observed in Caucasian CHD cases and not in African-Americans. The CHD cases in our study were of Caucasian origin, hence this could not explain the contrary findings of the both studies. However, due to the inclusion of patients who were exclusively from Central Europe and the resulting well defined demography it is assumed that the ethnical background of our study cohort is more homogenous than in the previously reported study.

Furthermore, in the reported study the mean age of the population was significantly younger (CHD cases: 55.8 ± 0.17 years, controls: 53.8 ± 0.10 years) than in the present study. This indicates that association of *EPHX2 K55R *may be only detectable in early-onset CHD. To review this hypothesis, the genotype distribution in CHD cases as well as in the control group was analysed with respect to the age of the patients. Our findings revealed no significant difference in the genotype distribution when only patients aged ≤ 55 years were analysed. This fact eliminates the possibility of an exclusive effect on early-onset CHD.

The fundamental difference of our study population compared to the previous study was the diagnostic inclusion criteria of the patients. In the present study all CHD patients had undergone a PCI with exclusion of cases suffering from acute coronary syndrome, whereas in the previously cited study more than half of all the patients suffered from acute myocardial infarction. The difference in patient characteristics may be the cause for the contrary findings.

Since several studies revealed an influence of *EPHX2 *gene expression on blood pressure by alterations in EET levels, the association of the *K55R *variant with increased risk of hypertension was investigated. Our findings showed no correlation between the *EPHX2 K55R *variant and decreased blood pressure in the studied population. Previous studies revealed that changes in blood pressure are accompanied with alterations in the epoxide enzyme activity. For instance, higher soluble epoxide hydrolase activity in carriers of at least one *K55R *variant allele was described in CHD patients of Caucasian origin [28]. In our study the enzyme activity of the soluble epoxide hydrolase and EET levels were not quantified because they where not defined as target parameters of this genetic study.

To our knowledge, the present study is the first to investigate the effect of the *EPHX2 K55R *polymorphism on the occurrence of restenosis after primary successful PCI. However, not even an association with incident CHD could be confirmed.

Certainly, the value of our results is limited due to the relatively low number of analysed individuals. Assuming a case-control design and type I error α = 0.05, we had a power of 0.47 to detect proportions of the *K55R *variant allele of 20.8% in CHD cases and 15.3% in the controls derived from the previous report [28]. It has to be noted that, in genome wide scans of large patient cohorts, no association was found between CHD and the locus of *EPHX2 *on chromosome 8p21-p12 [36]. This fact supports the findings of the present study.

## Conclusion

The results of the present study show no significant association of the *EPHX2 K55R *allele variant with the development of restenosis in patients over a period of six month after PCI. Furthermore, the association of this genetic variant with incident CHD could not even confirmed.

In conclusion, the present results suggest that the *EPHX2 K55R *polymorphism can be excluded as independent predictor for coronary heart disease or for the occurrence of restenosis in patients who had undergone primary successful PCI.

## Competing interests

The authors declare that they have no competing interests.

## Authors' contributions

SK established and performed the genotyping, analyzed the data and drafted the manuscript. PB administrated the blood samples and the clinical data. KR performed the DNA preparation and sample management. AH critically evaluated the statistics. GH, ML and RF revised the phenotype data and the manuscript. HPB designed and coordinated the clinical study and performed the enrolling and phenotyping of the patients. EvH provided the blood samples and the patients baseline characteristics of the controls. TS designed the genetic studies, made adjuvant contributions to the interpretation of the results and critically revised the manuscript. All authors have read and approved the final manuscript.

## Pre-publication history

The pre-publication history for this paper can be accessed here:


